# The perceived COVID-19 pandemic risk and mental distress in China: the mediating role of interpersonal trust and the moderating role of social cohesion

**DOI:** 10.3389/fpsyg.2025.1651664

**Published:** 2025-09-25

**Authors:** Hao Zhou, Xiangqian Liu, Jing Wu, Chuanjing Liao, Shengyu Zhao

**Affiliations:** 1 Moral and Psychological Education Research Center, Zhejiang University of Finance and Economics, Hangzhou, China; 2 Department of Early Childhood Education, Faculty of Education, Shenzhen Polytechnic University, Shenzhen, China; 3 College of Education, Wenzhou University, Wenzhou, China; 4 School of Public Basic Studies, Wenzhou Polytechnic, Wenzhou, China

**Keywords:** COVID-19 pandemic, perceived risk, interpersonal trust, social cohesion, mental distress

## Abstract

**Introduction:**

An increasing number of studies have highlighted the consequences of the coronavirus disease 2019 (COVID-19) pandemic and the mechanisms through which it inflicts harm. However, few have examined the relationship between perceived pandemic risk and mental distress from an interpersonal perspective. Drawing on the stress system model, trust theory, social-support theory, and exchange–emotion–cohesion theory, the present study investigates whether perceived pandemic risk is positively associated with mental distress, whether different types of interpersonal trust mediate this relationship, and whether social cohesion moderates this mediating process.

**Methods:**

The theoretical model was tested using data from 18,278 Chinese residents (Mage = 32.63 years, standard deviation = 13.85) between March and June 2020. Participants in this cross-sectional study completed a named survey assessing their perceived pandemic risk, different types of interpersonal trust, mental distress, and social cohesion. Correlational and moderated mediation analyses examined how perceived risk relates to mental distress via interpersonal trust, while estimating social cohesion’s moderated role in this pathway.

**Results:**

Perceived pandemic risk was positively associated with mental distress. Interpersonal trust among non-strangers (family, friends and colleagues) partially mediated this link, and social cohesion moderated the trust-to-distress pathway specifically for friends and colleagues trust. In contexts of lower social cohesion, the indirect effect through friends and colleagues trust was stronger, indicating greater vulnerability to mental distress.

**Discussion:**

These findings extend research on pandemic-related mental distress by identifying acquaintance trust in ordinary friends and colleagues as a key interpersonal mechanism and demonstrating that cohesive community contexts can buffer the psychological consequences of eroded trust. Practitioners should consider leveraging social relationships in public-health crisis responses.

## 1 Introduction

The global public health emergency caused by the coronavirus disease 2019 (COVID-19) pandemic has presented unprecedented challenges to residents in affected areas, garnering widespread international attention. As of October 31, 2022, there have been 83,178,070 confirmed cases of COVID-19, with 1,814,649 deaths reported (World Health Organization, 2022). Beyond the immense socioeconomic costs and loss of life associated with disease prevention and treatment, deeper issues have gradually come to light, among which mental health stands out. In stressful situations, damage to mental health not only occurs immediately (Holmes et al., 2020; World Health Organization, 2022) but also tends to develop and accumulate chronically, leading to what is known as the “whiplash effect” (Ashby et al., 2022; Qi et al., 2021; Sharma et al., 2024). For individuals in pandemic areas, living under the constant threat of disease can make them more susceptible to anxiety, fear, and depression compared to normal living conditions (Ren et al., 2020; Ahmed et al., 2024). Moreover, prolonged isolation and movement restrictions can trigger more severe psychological problems and mental disorders, including self-harm and suicidal ideation or behavior (Chen et al., 2023; Galea et al., 2020; Brooks et al., 2020). Unlike in ordinary times, mental distress that arise during special periods like the pandemic have their own distinct characteristics; they affect a larger number of people and are characterized by collectivity, universality, and suddenness (Brooks et al., 2020; Kim et al., 2024). Additionally, they can lead to a range of specific post-traumatic stress disorders (Hu et al., 2022; Lotzin et al., 2022; van Duinkerken et al., 2023). Therefore, investigating the contributing factors and underlying mechanisms of mental distress that emerge during pandemics is of significant importance for guiding effective non-physical defenses and constructing behavioral immune systems among the public during public health emergencies.

Early COVID-19 studies in China emphasized individual-level traits as determinants of residents’ negative affect (Osimo et al., 2021; Wang et al., 2020), while the mechanisms linking specific mental distress to interactive group relations received comparatively little attention. In practice, the provision of safe and effective interpersonal support remains a critical strategy for alleviating public psychological distress. Covering different events ranging from the 2003 severe acute respiratory syndrome outbreak and the 2014 Ebola pandemic to the 2015 spread of the Zika virus, numerous studies have emphasized the positive role of mutually supportive social relationships in pandemic prevention and control (Kuper et al., 2019; Deurenberg-Yap et al., 2005; van der Weerd et al., 2011; Yamanis et al., 2016; Trapido, 2019), such as how the disruption of community interactions can lead to public panic and interpersonal alienation (Blair et al., 2017; Vinck et al., 2019). Trust, as an important form of social capital for maintaining life satisfaction, has been proven to be significantly associated with individual well-being (Helliwell et al., 2014). From the initial outbreak to the ongoing spread of the pandemic, the loss of active interpersonal communication for self-protection can undermine the sense of trust between people (Bargain and Aminjonov, 2020), bringing multiple levels of instability to policy-based pandemic control (Fetzer et al., 2020). In addition, previous research has shown that community residents embedded in “proximity” social ties engage more readily in community activities, adapt better to their environment, and report greater equity, prosperity, and happiness, whereas those lacking such ties exhibit markedly lower life satisfaction (Perkins and Long, 2002). According to Putnam’s social capital theory (Putnam, 2000) and Xiang’s view on “the disappearance of the nearby” (Xiang, 2021), the characteristics of mutual assistance and coordination provided by community relationships can help individuals obtain greater survival support when facing difficulties. Therefore, with the loss of interpersonal trust caused by the pandemic, the social cohesion that residents’ communities have accumulated becomes particularly important (Hu et al., 2022), as it can buffer the negative impacts of physical and psychological isolation between people and provide more possibilities for community interaction. By integrating multiple theoretical frameworks, this study examines the social-interpersonal mechanisms through which perceived COVID-19 risk influences mental distress in the Chinese context. It offers a coherent framework for understanding how social capital operates during public-health crises and lays the groundwork for future research on interpersonal dynamics in disaster settings.

### 1.1 Perceived pandemic risk and mental distress

When individuals face adversity, stress and strain are natural psychophysiological responses, as people actively strive to reduce their stress levels to cope with greater levels of trauma (Lazarus and Folkman, 1984; Holmes and Rahe, 1967). From General Adaptation Syndrome (Selye, 1956) and the Stress and Coping Theory (Lazarus and Folkman, 1984) to the Stress-System Model for Functional Somatic Symptoms (Jiang, 2020), these models all indicate that changes in life events can lead to an imbalance in an individual’s psychophysiological system, thereby generating stress responses. During sudden disasters, for example, unexpected threats in the external living environment can cause extreme potential trauma, directly or indirectly harming people’s physical and mental health (First, 2024; Holmes and Rahe, 1967). In fact, consistent with the above views, previous studies have found that, during sudden public health events, including the COVID-19 pandemic, when people perceive the presence of pandemic risks in their living environment, it consumes the psychophysiological resources of individuals in the stress process, leading to various mental distress, including negative impacts caused by direct and vicarious trauma (Kira et al., 2021; Mak et al., 2009; Pfefferbaum and North, 2020). Among these, environmental change cues in life events play an important role in conveying danger information, including from both the natural physical environment and the human social environment, which can make residents perceive their own insecurity, increase uncontrollable uncertainty in the environment, and thereby affect mental health (Clayton, 2020; Lindell et al., 2017; Prati et al., 2012). For example, During an epidemic, increased proximity to confirmed cases, compounded by repeated isolations and mobility restrictions, heightens residents’ exposure to non-safe information, intensifies perceived pandemic risk, and markedly disrupts daily life (Garfin et al., 2020; Wei, 2020). At the same time, from a temporal perspective, perceived pandemic emergence, on the one hand, has a higher probability of causing more residents to develop acute stress disorder in the early stages. Some individuals, when facing environmental demands that are unpredictable and beyond their natural regulatory capacity, will exhibit non-specific responses such as fear, anxiety, hypochondria, depression, compulsion, and significant somatic symptoms, such as fatigue or sleep disturbances (Dou et al., 2022; Heanoy and Brown, 2024). On the other hand, during the sustained period, cumulative stress, referring to the cumulative effect of a series of stress events experienced by some individuals over the long term, which has a negative impact on individual health and well-being, also mounts (Yao et al., 2023). In confronting an pandemic, whether sudden or sustained, the perception of danger indeed causes mental distress at various time points. Based on the above literature, this study will examine mental distress during an ongoing pandemic by focusing on the perceived risk generated by proximity to infection sources. It is reasonable to hypothesize the following:

*Hypothesis 1*: The perceived risk of COVID-19 will be positively correlated with mental distress.

### 1.2 The mediating role of interpersonal trust

The reduction of interpersonal trust may be another potential consequence of a major pandemic. According to Ross’s (2011) coexistence perspective in trust theory, the loss of trust in others stems from an individual’s aversion to actual or potentially threatening environments when they feel a loss of control, serving as a self-protective response. In fact, from an evolutionary standpoint, individuals tend to adjust their behavioral strategies in high-risk, uncertain environments to minimize potential harm, which is a result of the behavioral immune system (Hintze et al., 2015; Makhanova and Shepherd, 2020). When people perceive actual insecurity in a disaster environment, due to limited resources, they may believe that the presence of others directly or indirectly threatens their survival, with a loss of trust being a consequence (Ross, 2011). Consistent with this view, empirical studies have shown that the severity of panic caused by disaster environments is positively correlated with low interpersonal trust (Aldrich and Meyer, 2015). Specifically, the COVID-19 pandemic was a sudden and enduring natural and social disaster that changed the environment (Miao et al., 2020). Due to the need for a sense of security, residents in pandemic areas remain highly vigilant toward their surroundings, including other people, during such events (Lee et al., 2023; Li et al., 2020; Mazza et al., 2020). Previous research has further found that the level of pandemic risk is negatively correlated with social trust (Ridenhour et al., 2022; Van Bavel et al., 2020). Moreover, in a sociocultural context like China, where relational hierarchies shape social interactions, trust is differentiated based on relational proximity, with stronger trust typically extended to family, followed by neighbors, acquaintances, and least to strangers (Fei, 1992). These gradations align with social capital theory, which posits that trust in “nearby” relationships (e.g., family, neighbors) provides greater survival support during crises (Putnam, 2000). Consequently, perceived pandemic risk is hypothesized to correlate negatively with trust in family, neighbors, and acquaintances due to heightened vigilance against person-to-person transmission, while trust in strangers may be less affected due to limited interaction.

The erosion of interpersonal trust may have a negative impact on individual mental health. According to social support theory (Cohen and Wills, 1985; Kim et al., 2008), trust is a crucial prerequisite for mutual support. When individuals trust others, they are more likely to seek and receive social support, which can alleviate stress, facilitate emotional comfort, and provide practical assistance, thereby promoting mental health (Wang et al., 2018). Individuals who lose trust in others often lack support and help from others in real life and struggle to meet their social interaction needs (Almakaeva et al., 2018). In fact, when facing disaster events, residents who trust each other can help one another, which gives individuals a greater sense of control and the acquisition of this social resource reduces their fear of the disaster (Lam and Kuipers, 2019). Once this trust system collapses, however, these residents cannot mitigate the psychological stress caused by disaster threats, including an pandemic, by establishing cooperative and mutually supportive relationships with others (Prati and Mancini, 2021; Van Bavel et al., 2020). Although people can maintain a safe distance from sources of pandemic infection by reducing trust, long-term isolation may lead to more negative psychological states, including loneliness and depression (Brooks et al., 2020; Galea et al., 2020). Consistent with the above views, multiple empirical studies have shown that social trust is negatively correlated with mental distress in various environments (Cruwys et al., 2020; Fancourt et al., 2021; Rotenberg et al., 2023; Rutar et al., 2025). Additionally, a meta-analysis found that interpersonal trust is positively correlated with subjective well-being, an indicator of mental health (Bai et al., 2019). Based on the above theory and the empirical literature, we hypothesize a directional pathway from risk perception through trust to mental distress:

*Hypothesis 2*: Interpersonal trust will mediate the relationship between perceived pandemic risk and mental distress.

### 1.3 The moderating role of social cohesion

Both physical and social environmental factors can jointly influence individuals’ mental health status. According to the Relational Cohesion Theory and its derivative extensions (such as the Affect Theory of Social Exchange) (Lawler and Yoon, 1996; Lawler et al., 2000; Lawler et al., 2006), cohesive communities convert mutual support into positive affect and attachment, whereas low-cohesion settings yield fewer emotional gains (Dragolov et al., 2016; Drury et al., 2019). Although negative life events (e.g., pandemics and other public health events) are generally regarded as risk factors (Brooks et al., 2020), individuals in environments lacking social cohesion may perceive them as more severe issues. Compared to those in environments with high social cohesion, these individuals exhibit greater levels of stress responses to threats in their living environment (Li et al., 2023; Sonnenfeld et al., 2021; Svensson and Elntib, 2021). Moreover, these individuals are not only more prone to developing excessive anxiety, fear, and maladaptive behaviors (Hikichi et al., 2016; Kim, 2020) but also manifest somatic symptoms resulting from mental distress (Silveira et al., 2022). During some pandemic periods, residents in high social cohesion areas united by shared consensus and behavioral alignment more actively adhered to social norms such as mask wearing and vaccination, engaged in greater material and emotional mutual aid, and sustained stronger confidence in overcoming disasters, thereby effectively mitigating the psychological toll of the disaster (Cárdenas et al., 2023; Gibson and Losee, 2023; Townshend et al., 2015; Ware, 2024). Studies from pandemic areas in China indicate that residents in highly cohesive settings report elevated social support, perceived control, and security, which jointly buffer pandemic-related hostility, loneliness, anxiety, and fear (Bian et al., 2021; Nie et al., 2024; Qin, 2021). Therefore, it is reasonable to propose the following hypothesis:

*Hypothesis 3*: Residents facing high-risk pandemics in low-social cohesion contexts exhibit diminished interpersonal trust and elevated mental distress. Social cohesion may moderate the relationship between perceived pandemic risk and mental distress in three ways: (1) the direct link between perceived pandemic risk and mental health, (2) the link between perceived pandemic risk and interpersonal trust, and (3) the link between interpersonal trust and mental distress.

**Figure 1:**
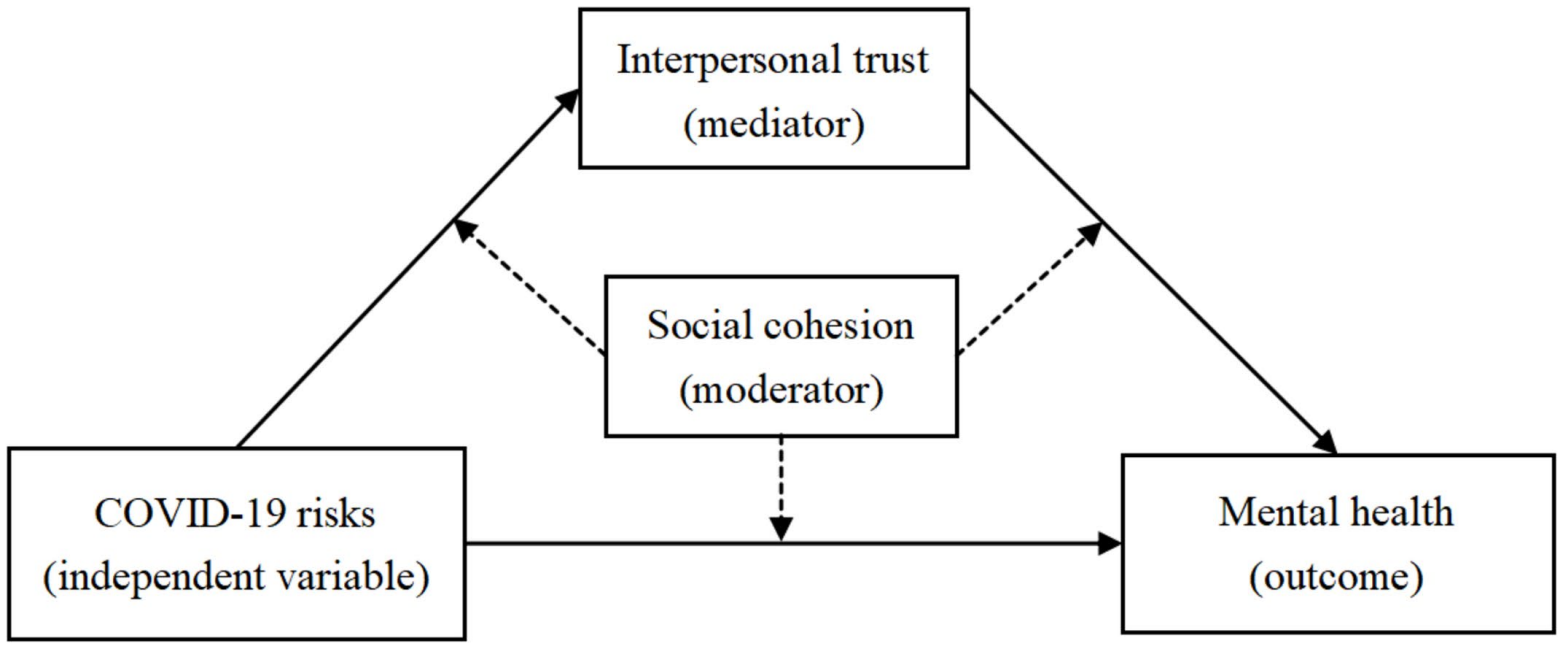
The proposed moderated mediation model.

In summary, this study proposes a moderated mediation model (see Figure 1) based on a series of stress-related theories, trust theory, social support theory, and relational cohesion theory to examine the associations between perceived COVID-19 risks and mental distress, as well as the mediating role of interpersonal trust and the moderating role of social cohesion.

## 2 Materials and methods

### 2.1 Participants

The initial sample of this study included 20,235 Chinese residents, with a response rate of 92.76%. Data cleaning involved removing 1,957 participants due to incomplete responses (missing more than 20% of questionnaire items, *n* = 1,342), inconsistent demographic data (e.g., reported age outside plausible range, *n* = 412), or duplicate submissions (*n* = 203). The final sample size was reduced to 18,278 individuals (10,484 females, 57.4%; 7,794 males, 42.6%; *M*_age_ = 32.63 years, standard deviation [*SD*] = 13.85, range = 15–100 years). After obtaining informed consent, participants completed a questionnaire that included demographic information, perceived COVID-19 risk, interpersonal trust, social cohesion, and mental distress.

### 2.2 Procedure

A survey was conducted from April 22 to April 28, 2020, across 31 provinces or provincial-level administrative regions in China, using an online questionnaire format. All the materials and procedures of this study were approved by the institutional review board of Zhejiang University of Finance Economics (ZUFEPSY-2020-04-001) prior to the recruitment of participants. Specifically, data collection was executed via an innovative protocol designed to ensure respondent engagement equivalent to that of face-to-face interviews: each surveyor first contacted prospective participants to secure informed consent, then delivered a personalized survey link and secure access code for self-administration. Unlike typical online surveys that are simply posted on online platforms with little knowledge of the respondents, this survey approach ensured meaningful engagement. After obtaining informed consent, research assistants from 19 universities, who had undergone training, conducted the survey according to a standardized script. The study ensured anonymity, and participants were free to withdraw from the study at any time without any negative consequences.

### 2.3 Measures

#### 2.3.1 Perceived COVID-19 risk

Perceived COVID-19 risk, defined as the subjective assessment of risk based on the proximity of confirmed infections, is a critical factor influencing mental distress during pandemics. This study referred to the “Ripple Effect” (Kasperson et al., 1988; Slovic, 1987; Wen et al., 2020; Xie and Lin, 2012) to assess the degree of pandemic risk perceived by residents. The survey scale consisted of five items asking respondents whether there were confirmed COVID-19 cases within their contact range. If respondents perceived that individuals in close contact with them were infected, they were considered to have a greater level of perceived infection risk. In detail, residents made corresponding judgments about the risk within different scopes of their social circles. All five items were scored using a forced-choice method (No = no cases, Yes = presence of cases), with scores increasing progressively based on the proximity of the relationship (0 = no cases, 1 = cases in the same community, 2 = cases among friends/classmates/colleagues, 3 = cases in the same building, 4 = cases among non-cohabitating family members, 5 = cases among cohabitating family members). The higher the final score, the greater the perceived risk of exposure to a COVID-19 case. For example, if a respondent had a cohabitating family member diagnosed with COVID-19, the highest score (5 points) was assigned, while individuals who reported no confirmed cases received the lowest risk score (0 points). Additionally, to avoid redundant scoring for different scopes, such as cohabitating family members, residents in the same building, or residents in the same community, the scale used the highest score among the items as the measure of perceived COVID-19 risk.

#### 2.3.2 Interpersonal trust

Consistent with the items tested in the General Trust Scale (Yamagishi et al., 2015) and Interpersonal Distrust Scale (Goldberg et al., 2006), this study directly selected the keyword “trust” to measure individuals’ interpersonal trust during the COVID-19 pandemic. Given the characteristics of the relational hierarchy in Chinese Confucian cultural traditions, where individuals have varying degrees of trust in different groups of people, especially the emphasis of Neo-Confucianism on the close relationship between interpersonal relationships and group attributes, the scale divided the trust targets into four main groups based on the spatial scope of Chinese residents’ lives: family members, friends and colleagues, neighbors, and strangers. Specifically, acquaintances represent frequent, role-based interactions (e.g., workplace or social circles), while neighbors reflect community-based, spatially proximate relationships critical for mutual support in Chinese urban and rural settings (Fei, 1992). Therefore, the scale included a total of four items, with the same question content but different subjects of investigation, such as “How much do you trust others (including family members, friends and colleagues, neighbors, strangers)?” All items were independently scored using a 4-point scale ranging from 1 (completely distrust) to 4 (extremely trust) points, with higher scores indicating greater levels of trust for each target.

#### 2.3.3 Social cohesion

Social cohesion was assessed using two items from the Neighborhood Cohesion Scale (Sampson et al., 1997; Sampson et al., 2002). Specifically, respondents in the pandemic areas were asked to rate the degree of social cohesion they experienced on a 5-point scale ranging from 1 (never) to 5 (always) points. For example, one item was “People in this neighborhood are willing to help their neighbors,” while another item was “People in this neighborhood are trustworthy.” The scores of the two items were averaged, with higher scores indicating a greater level of social cohesion in the individual’s living environment. In this study, the scale also demonstrated good reliability (Cronbach’s *α* = 0.789).

#### 2.3.4 Mental distress

Mental distress was assessed using five typical items from the Patient Health Questionnaire (PHQ-4) developed by Löwe et al. (2010) and the Center for Epidemiologic Studies Depression Scale (CES-D) developed by Radloff (1977) and revised by Hann et al. (1999). Both the PHQ-4 and CES-D are widely used in clinical and epidemiological studies to detect the level of psychological distress (Costantini et al., 2021; Hann et al., 1999; Kroenke et al., 2001; Rehm and Shield, 2019). In this study, participants read five items and reported the frequency of their feelings over the past week on a 5-point scale ranging from 1 (rarely) to 5 (always) points; example items include “feeling nervous or shaky inside,” “feeling afraid,” “feeling down,” “worrying too much about things,” and “feeling hopeless about the future.” Participants’ responses were summed, with total scores ranging from 5 to 25 points and higher scores indicating a poorer mental health status. In this study, the confirmatory factor analysis results showed that the scale had acceptable structural validity, with standardized root mean square residual (SRMR) = 0.04, comparative fit index (CFI) = 0.96, and Tucker–Lewis index (TLI) = 0.92. The scale also demonstrated good reliability (Cronbach’s *α* = 0.88).

### 2.4 Test process and data processing

Data analysis was conducted using SPSS version 26.0 (IBM Corporation, Armonk, NY, USA). Prior to statistical analysis, responses with missing data were excluded from data processing, as the proportion of missing data for all variables was very low (<2%). Subsequently, descriptive statistics and bivariate correlations were calculated for each variable. Next, Hayes’s PROCESS macro Model 4 (Hayes, 2013) was employed to examine the mediating role of interpersonal trust in the relationship between perceived pandemic risk and mental distress. The proportion of the total effect mediated was calculated as the indirect effect divided by the total effect (direct + indirect) for each mediator, expressed as a percentage (Hayes, 2013). Finally, Hayes’s PROCESS macro Model 59 (Hayes, 2013) was used to explore whether social cohesion moderated this mediating process. Given that gender (birth sex) and age are important predictors of mental health (Seedat et al., 2009) and typical confounders, they were included as control variables in all regression models. Before applying both models, all continuous variables (perceived COVID-19 risk, interpersonal trust, social cohesion, and psychological distress) were z-standardized to ensure comparability. Gender was coded as a dummy variable (0 = female, 1 = male) and included as a covariate. Interaction terms for the moderation analysis were calculated using the standardized values of interpersonal trust and social cohesion. Bias-corrected percentile bootstrapping (with 5,000 resamples) was used to determine the significance of the effects.

## 3 Results

### 3.1 Common method bias test

During the survey administration process, this study controlled for potential common method bias procedurally and used the Harman single-factor test to assess the degree of common method variance. Setting the number of common factors to 1, the confirmatory factor analysis results showed poor model fit, where SRMR = 0.12, CFI = 0.61, and TLI = 0.55. Therefore, common method bias was not significant in this study.

### 3.2 Preliminary analysis

The descriptive statistics for the demographic information of the respondents are presented in Table 1. As expected, the interviewees were widely distributed, and the survey characteristics were extensive, which fully demonstrates the validity and representativeness of the sample. The correlation matrix (zero-order Pearson’s correlation coefficients) is shown in Table 2. As expected, perceived pandemic risk is significantly positively correlated with mental distress (*r* =0.045, *p* <0.01). It is also significantly negatively correlated with family trust (*r* = −0.043, *p* < 0.01), neighbor trust (*r* = −0.015, *p* < 0.05), and acquaintance trust (*r* = −0.023, *p* < 0.01), while the negative correlation with stranger trust (*r* = 0.007, *p* > 0.05) is not significant. Additionally, family trust (*r* = −0.168, *p* < 0.01), neighbor trust (*r* = −0.133, *p* < 0.01), acquaintance trust (*r* = −0.110, *p* < 0.01), and stranger trust (*r* = 0.032, *p* < 0.01) are all significantly negatively correlated with mental distress. Furthermore, social cohesion is not significantly negatively correlated with perceived pandemic risk (*r* = −0.007, *p* > 0.05) but is significantly negatively correlated with mental distress (*r* = −0.108, *p* < 0.01). It is also significantly positively correlated with family trust (*r* = 0.142, *p* < 0.01), neighbor trust (*r* = 0.368, *p* < 0.01), acquaintance trust (r = 0.242, *p* < 0.01), and stranger trust (*r* = 0.157, *p* < 0.01). A post-hoc sensitivity analysis using G*Power indicated that, with a sample size of 18,278, *α* = 0.05, and power = 0.80, the study could reliably detect a correlation as small as *r* = 0.018, confirming sufficient power to detect the small effect sizes observed in the mediation and moderation analyses. Given that perceived pandemic risk is positively correlated with mental distress, Hypothesis 1 is supported.

**Table 1:** Descriptive statistics of the analytical sample (*N* = 18,278).

Variables	Mean/Percentage	Std. Dev
Age (years)	32.633	13.854
Male	42.641%	
College education	48.889%	
Income	3.072	1.549
Married	44.945%	
Household size	4.013	1.398

The monthly income ranged from 1 to 7 (1 = 0–2000; 2 = 2001–5,000; 3 = 5,001–8,000; 4 = 8,001–10,000; 5 = 10,001–20,000; 6 = 20,001–30,000; 7 = 30,000+).

**Table 2:** Descriptive statistics and correlations among variables.

	M ± SD	1	2	3	4	5	6	7
1 Perceived risk	0.22 ± 0.82	1						
2 Trust (family)	3.71 ± 0.51	−0.043^**^	1					
3 Trust (neighbors)	2.96 ± 0.52	−0.015^*^	0.227^**^	1				
4 Trust (f & c)	3.07 ± 0.43	−0.023^**^	0.254^**^	0.588^**^	1			
5 Trust (strangers)	2.08 ± 0.72	−0.007	−0.032^**^	0.337^**^	0.283^**^	1		
6 Mental distress	1.40 ± 0.58	0.045^**^	−0.168^**^	−0.133^**^	−0.110^**^	−0.032^**^	1	
7 Social cohesion	3.60 ± 0.70	−0.007	0.142^**^	0.368^**^	0.242^**^	0.157^**^	−0.108^**^	1

*N* = 18,278. f & c, friends and colleagues (acquaintance). **p < 0.01; *p < 0.05.

### 3.3 Testing for the mediating role of interpersonal trust

Consistent with Hypothesis 2 and the correlations among variables, interpersonal trust at various levels (except for trust in strangers) mediated the relationship between perceived pandemic risk and mental distress. This was tested using Hayes’s (2013) PROCESS macro Model 4. The results showed that perceived pandemic risk was negatively correlated with family trust (*b* = −0.044, *p* < 0.001; see Model 2a in Table 3), acquaintance trust (*b* = −0.024, *p* = 0.001 < 0.01; see Model 2b in Table 4), and neighbor trust (*b* = −0.016, *p* = 0.029 < 0.05; see Model 2c in Table 5). Additionally, family trust (*b* = −0.162, *p* < 0.001; see Model 3a in Table 3), acquaintance trust (*b* = −0.107, *p* < 0.001; see Model 3b in Table 4), and neighbor trust (*b* = −0.123, *p* < 0.001; see Model 3c in Table 5) were negatively correlated with distress. The direct association between perceived pandemic risk and mental distress was also significant (*b* = 0.046, *p* < 0.001; see Model 1 in Tables 3–5). The bias-corrected percentile bootstrapping results indicated significant indirect effects through family trust (indirect effect = 0.007, standard error [*SE*] = 0.002, 95% confidence interval [*CI*] = [0.004, 0.010], proportion mediated = 15.434%), acquaintance trust (indirect effect = 0.003, *SE* = 0.01, 95% *CI* = [0.000, 0.005], proportion mediated = 5.652%), and neighbor trust (indirect effect = 0.002, *SE* = 0.001, 95% *CI* = [0.000, 0.004], proportion mediated = 4.348%). However, to ensure the robustness and reliability of the results, we applied Bonferroni correction to adjust the significance level (*p_adjust_* = 0.05/3 = 0.017). Consequently, the indirect effect via neighbor trust fails to exceed the corrected threshold. Therefore, two types of interpersonal trust (family, acquaintance) mediated the relationship between perceived pandemic risk and mental distress, with family trust having the strongest mediating effect. This partially supports Hypothesis 2.

**Table 3:** Testing the mediation effect of perceived risk on mental distress via trust (family).

Predictor	Model 1 mental distress		Model 2a trust (family)		Model 3a mental distress	
	*b*	*t*	*b*	*t*	*b*	*t*
Perceived risk	0.046	6.249^***^	−0.044	−5.970^***^	0.039	5.345^***^
Trust (family) trust					−0.162	−22.240^***^ 3.995^*^
Gender	0.052	3.478^***^	−0.043	−2.891^**^	0.045	3.049^**^
Age	−0.006	−11.897^***^	0.003	6.157^***^	−0.006	−11.032^***^
*R* ^2^	0.011		0.005		0.037	
*F*	66.437^***^		28.086^***^		1745.833^***^	

*N* = 18,278. Each column is a regression model that predicts the criterion at the top of the column. ^***^p < 0.001; ^**^p < 0.01; ^*^p < 0.05.

**Table 4:** Testing the mediation effect of perceived risk on mental distress via trust (f & c).

Predictor	Model 1 mental distress		Model 2b trust (f & c)		Model 3b mental distress	
	*b*	*t*	*b*	*t*	*b*	*t*
Perceived risk	0.046	6.249^***^	−0.024	−3.103^**^	0.043	5.932^***^
Trust (f & c) trust					−0.107	−14.667^***^
Gender	0.052	3.478^***^	−0.070	−4.647^***^	0.045	2.993^**^
Age	−0.006	−11.897^***^	0.000	0.696	−0.006	−11.891^**^
*R* ^2^	0.011		0.002		0.022	
*F*	66.437^***^		10.907^***^		104.195^***^	

*N* = 18,278. Each column is a regression model that predicts the criterion at the top of the column. ^***^p < 0.001; ^**^p < 0.01; ^*^p < 0.05. f & c, friends and colleagues (acquaintance).

**Table 5:** Testing the mediation effect of perceived risk on mental distress via trust (neighbors).

Predictor	Model 1 mental distress		Model 2c trust (neighbors)		Model 3c mental distress	
	*b*	*t*	*b*	*t*	*b*	*t*
Perceived risk	0.046	6.249^***^	−0.016	−2.185^*^	0.044	6.025^***^
Trust (neighbors)					−0.123	−16.736^***^
Gender	0.052	3.478^***^	−0.068	−4.599^*^	0.043	−2.933^**^
Age	−0.006	−11.897^***^	0.007	13.958^***^	−0.005	−10.205^**^
*R* ^2^	0.011		0.013		0.026	
*F*	66.437^***^		77.608^***^		120.611^***^	

*N* = 18,278. Each column is a regression model that predicts the criterion at the top of the column. ^***^p < 0.001; ^**^p < 0.01; ^*^p < 0.05.

### 3.4 Testing for moderated mediation

**Figure 2:**
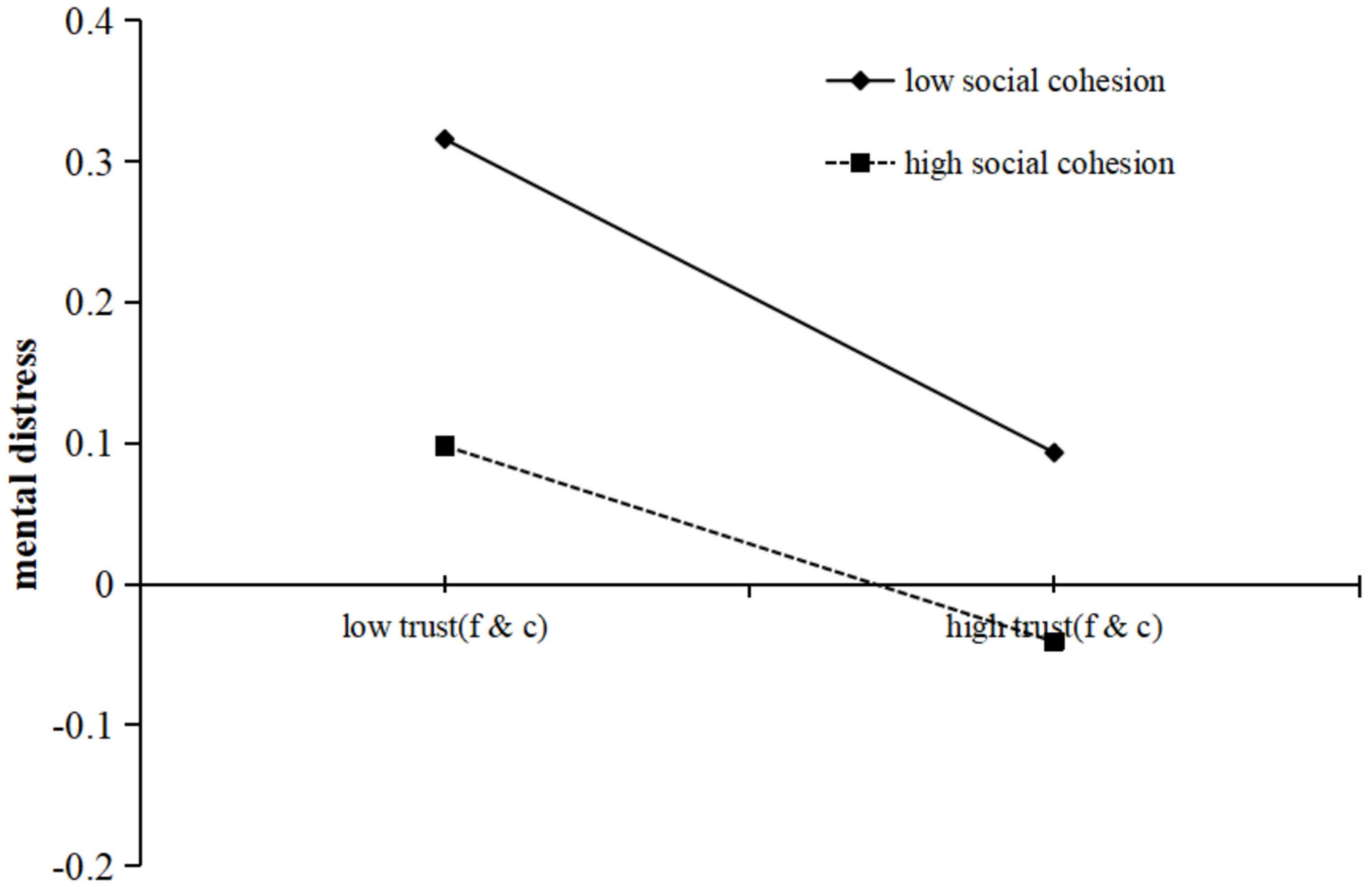
Social cohesion moderates the relationship between trust (friends and colleagues) and mental distress.

Hypothesis 3 predicted that social cohesion would moderate the relationship between perceived pandemic risk and mental distress, including all three paths. To test this hypothesis, Hayes’s (2013) PROCESS macro Model 59 was employed. The results indicated that social cohesion did not moderate any of the paths in the family trust–mediation model (see Table 6). However, it significantly moderated the second stage of the indirect effects through acquaintance trust (*b* = 0.022, *p* < 0.001; see Table 7). This statistical findings achieved the Bonferroni-adjusted significance criterion (*p_adjust_* = 0.05/2 = 0.025), attesting to their reliability. To more clearly illustrate this moderating effect, we plotted the impact of acquaintance trust (Figure 2) on mental distress for participants with low and high social cohesion (1 *SD* below and 1 *SD* above the mean, respectively). The simple slope tests revealed that, among pandemic area residents in environments of low social cohesion, mental distress significantly increased as acquaintance trust declined (*b*_simple_ = −0.113, *t* = −11.381, *p* < 0.001). Conversely, among those in environments of high social cohesion, the increase in mental distress was more tempered as acquaintance trust decreased (*b*_simple_ = −0.068, *t* = −7.252, *p* < 0.001). This further highlights the pronounced moderating effect of social cohesion in communities characterized by lower cohesion.

**Table 6:** Testing the moderated mediation effect of trust (family) on mental distress.

Predictor	Model 1 trust (family)		Model 2 mental distress	
	*b*	*t*	*b*	*t*
Perceived risk	−0.043	−5.864^**^	0.039	5.341^***^
Trust (family)			−0.151	−20.166^***^
Social cohesion	0.138	18.733^***^	−0.078	−10.546^***^
Risk*SC	0.001	0.075	0.008	1.040
Trust (family)*SC			−0.000	−0.022
Gender	−0.036	−2.428^*^	0.041	2.800^*^
Age	−0.002	4.260	−0.005	−9.971^***^
*R* ^2^	0.023		0.043	
*F*	87.357^***^		116.564^***^	

*N* = 18,278. Each column is a regression model that predicts the criterion at the top of the column. ^***^p < 0.001; ^**^p < 0.01; ^*^p < 0.05. SC, social cohesion.

**Table 7:** Testing the moderated mediation effect of trust (f & c) on mental distress.

Predictor	Model 1 trust (f & c)		Model 2 mental distress	
	*b*	*t*	*b*	*t*
Perceived risk	−0.022	−3.045^**^	0.043	5.914^***^
Trust (f & c)			−0.091	−12.013^***^
Social cohesion	0.243	33.640^***^	−0.079	−10.383^***^
Risk*SC	0.004	0.531	0.009	1.212
Trust (f & c)*SC			0.022	3.697^***^
Gender	−0.057	−3.910^***^	0.045	2.993^**^
Age	−0.001	−2.733	−0.006	−11.891^**^
*R* ^2^	0.060		0.029	
*F*	233.336^***^		76.785^***^	

*N* = 18,278. Each column is a regression model that predicts the criterion at the top of the column. ^***^p < 0.001; ^**^p < 0.01; ^*^p < 0.05. f & c, friends and colleagues; SC, social cohesion.

The indirect association between perceived pandemic risk and mental distress via acquaintance trust was stronger among residents with lower social cohesion experiences (indirect effect = 0.003, *SE* = 0.002, 95% *CI* = [0.000, 0.006]) than those with higher social cohesion experiences (indirect effect = 0.001, *SE* = 0.001, 95% *CI* = [0.000, 0.003]). The index of moderated mediation was significant (difference between the two indirect effects = 0.002, *SE* = 0.001, 95% *CI* = [0.000, 0.006]). Therefore, Hypothesis 3 was partially supported.

## 4 Discussion

The COVID-19 pandemic inflicted substantial losses on nations and their populations across the world, akin to other catastrophic events. Within the initial 6-month period, the global economic fallout from the pandemic was estimated to reach a staggering $8.5 trillion (International Monetary Fund, 2020; United Nations, 2020). Extensive research has been conducted on the mechanisms for responding to a pandemic, with a particular focus on policy implementation, organizational infrastructure, economic assistance, and medical support (Carter and May, 2020; Hale et al., 2021; Li et al., 2021; Saputro and Prakoso, 2021; Zhang et al., 2022). However, compared to physical defenses, research on non-physical defenses—specifically, mental health protection mechanisms—has remained relatively underdeveloped. China, being the region with the largest population affected by the pandemic, necessitates a heightened focus on the mental health issues of its populace, encompassing both acute psychological stress and chronic psychological trauma. Grounded in a series of stress-related theories (Holmes and Rahe, 1967; Jiang et al., 2022; Lazarus and Folkman, 1984), trust theory (Ross, 2011), social support theory (Cohen and Wills, 1985; Kim et al., 2008), and relational cohesion theory (Lawler and Yoon, 1996; Lawler et al., 2000; Lawler et al., 2006), this study specified a moderated mediation model to test whether perceived pandemic risk is indirectly associated with mental distress among residents in pandemic areas through diminished trust, and whether social cohesion moderates this indirect association. All findings extend prior theories by highlighting the differential roles of trust types in a pandemic context and underscore the protective role of social cohesion in mitigating distress.

### 4.1 Perceived pandemic risk and mental distress

The study’s findings revealed a negative correlation between perceived pandemic risk and the mental distress of residents in pandemic areas, indicating that higher perceived threat levels are associated with a greater incidence of mental health issues, thereby supporting Hypothesis 1. While existing research has extensively explored the stress-related responses, such as anxiety, fear, and depression, induced by the spread of various infectious diseases (Mak et al., 2009; Novihoho et al., 2022; Substance Abuse and Mental Health Services Administration (SAMHSA), 2014), large-scale and prolonged outbreaks like the current pandemic are rare. Consequently, studies examining the physical isolation, media campaigns, and safety monitoring measures associated with such catastrophic pandemic responses are also scarce (Hale et al., 2021; World Health Organization, 2022; World Health Organization, 2025; Zhang et al., 2022).

In fact, beyond the direct threat to life (infection, lack of medical treatment) revealed by previous studies, the physical isolation and the uncertainty of safe time and space in the environment caused by the pandemic can also lead to various psychological problems (Brooks et al., 2020; Sharma et al., 2024; Wei, 2020). Especially in the early stages of an pandemic, the sense of “unknown” can cause widespread panic and helplessness among the public, which in turn threatens social stability and the implementation of public health policies (Dailey, 2024; Svensson and Elntib, 2021). For example, a meta-analysis found that higher perceived pandemic risk in the early stages of an pandemic is significantly positively correlated with negative emotions (such as anxiety, tension, depression, fatigue, loneliness, and boredom), and this perception directly affects the mental distress of residents (Zhou et al., 2024). Therefore, given the heightened vigilance of individuals to the sources of danger in their environment, this stress perception directly impacts the mental distress of residents (Lotzin et al., 2022; Ren et al., 2020), and the severity of life-threatening risks may be positively correlated with behavioral immune effects (Yang et al., 2020) and various mental distress indicators (Holmes et al., 2020). Building on previous research, we further focus on the relationship between the threat perception of this major pandemic and the mental distress of residents, which is consistent with the expectations of various stress theories proposed by predecessors and aligns with the perspectives of behavioral biology and environmental models (Ackerman et al., 2018; Hansen et al., 2022). This indicates that not only direct illness or infection but also the mere perception of nearby viral threats, high proximity to the source of infection, or even just contact with strangers and exposure to deadly information can impact an individual’s mental state (Kämpfen et al., 2020).

### 4.2 The mediating role of interpersonal trust

This study’s findings extend pre-pandemic disaster mental health research by highlighting the unique role of interpersonal trust and social cohesion during the COVID-19 pandemic. Unlike natural disasters such as hurricanes or earthquakes, where physical proximity fosters collective coping (Kaniasty and Norris, 2004), the pandemic’s social distancing measures disrupted direct social interactions, amplifying the mediating role of trust in family and acquaintances. Consistent with our Hypothesis 2, interpersonal trust at multiple levels mediated the relationship between perceived pandemic risk and mental distress. Specifically, the perception of pandemic risk indirectly affects individuals’ mental distress through their trust in various social relationships (Ross, 2011; Schaller et al., 2021; Worchel, 1979), corroborating the functioning of the behavioral immune system in uncertain environments (Makhanova and Shepherd, 2020; Shook et al., 2020) and the social support effects of trust (Cohen and Wills, 1985; Kim et al., 2008). In fact, this study consistently examined the relationship between pandemic threat perception and mental distress from an interpersonal perspective, providing potential reasons for this phenomenon. Relatively speaking, researchers rarely encounter a pandemic of this magnitude and are able to empirically test this relationship or investigate the underlying mechanisms through large-scale surveys (Lee et al., 2023; World Health Organization, 2022; World Health Organization, 2025).

Beyond the overall effect, each independent link within the mediation process warrants attention, with different levels of interpersonal trust exhibiting distinct mechanisms. Regarding the link between perceived pandemic risk and interpersonal trust, the results partially supported the notion that high levels of pandemic threat perception, as a form of stress response (Holmes and Rahe, 1967; Jiang et al., 2022), can lead to a decrease in interpersonal trust (Ross, 2011; Ridenhour et al., 2022). Importantly, the significant impact is on trust in familiar others, not on trust in strangers. Although establishing supportive trust relationships in high survival pressure environments has always been one of the main tasks for pandemic area residents to protect their physical and mental health (Fell, 2020; Miao et al., 2020), when individuals perceive more life-threatening risks from “person-to-person transmission” during the pandemic, they may develop distrustful attitudes and behaviors toward others around them (Fang et al., 2023; Thoresen et al., 2021). In fact, interpersonal trust involves positive expectations of others’ behavior and a willingness to rely on others or systems in the face of uncertainty and potential risks (Worchel, 1979; Yuan et al., 2022). Looking specifically at the study results, compared to strangers, family, neighbors friends and colleagues constitute the primary relational targets with whom individuals maintain the closest and most frequent interactions and upon whom they rely most heavily. Consequently, increasing local COVID-19 incidence elevates perceived infection risk via these ties. Driven by the instinct to protect their own safety, this uncertain life-threatening environment led individuals to develop a heightened sense of danger, reduce positive expectations of others, and decrease reliance on each other (Fang et al., 2023; Han and Curtis, 2021). Notably, in contemporary societies marked by the erosion of spatial proximity and heightened social atomization, neighborly interactions have atrophied, increasingly resembling the fleeting and impersonal encounters characteristic of strangers (Putnam, 2000; Xiang, 2021). This finding particularly highlights the importance of not overlooking the temporary decline in trust within individuals’ “familiar interpersonal” systems during disasters. External interventions are needed to recreate the previously supportive atmosphere of mutual dependence in the environment, which can help buffer a series of negative outcomes following adverse events (Chan, 2021).

Regarding the relationship between interpersonal trust and mental distress, the results indicated that individuals’ trust in others was negatively correlated with their mental distress, consistent with prior research (Cruwys et al., 2020; Rotenberg et al., 2023; Rutar et al., 2025). Specifically, in the face of severe infectious diseases, people chose to keep a distance from others to minimize perceived risk, which was accompanied by a loss of trust in others (Jiang et al., 2009; Ridenhour et al., 2022). This decline in trust signifies the loss of a robust social support system, which in turn affects physical and mental health (Prati and Mancini, 2021; Van Bavel et al., 2020). Numerous studies have shown that interpersonal trust, as a crucial component of social capital, plays an indispensable role in maintaining individual mental health (Bai et al., 2019; Fancourt et al., 2021). Against the backdrop of the pandemic, individuals lacking interpersonal trust may experience increased psychological distress due to feelings of isolation and helplessness (Billah et al., 2023; Prati and Mancini, 2021), while those in trusting environments can gain emotional support and other social resources, effectively buffering the stress brought on by negative events (Costanza et al., 2021). Thus, the study results support the theoretical expectations of the “social capital” model (Coleman, 1988; Putnam, 1995). In other words, “interpersonal trust,” as a vital form of social capital, can provide a more robust safety net for the physical and mental health of individuals in pandemic areas.

### 4.3 The moderating role of social cohesion

Compared with major pandemic conditions, pre-pandemic studies reported more readily available physical community support (Kaniasty and Norris, 2004), thereby highlighting the distinctive social dynamics of the pandemic itself. Specifically, this study’s findings revealed that social cohesion did not significantly moderate the relationship between perceived pandemic risk and various types of interpersonal trust, nor the relationship between perceived pandemic risk and mental distress. This finding aligns with some scholars’ views that, when confronted with a pandemic, even if mutual assistance behaviors and expectations persist, the psychological states of ordinary people are still more uncontrollably damaged than imagined, including their trust in others (Fang et al., 2023; Han and Curtis, 2021; Thoresen et al., 2021). In contrast, social cohesion did moderate the association between acquaintance trust and mental distress, partially supporting Hypothesis 3. This result is consistent with the notion that social cohesion places greater emphasis on the “mutual support” in individuals’ living environments rather than on closest family interaction settings or distant stranger interaction settings (Putnam, 2015; Unger and Wandersman, 1985). Specifically, pandemic area residents with greater perceived social cohesion in their living environment exhibited stronger associations between acquaintance trust and mental distress, which is an effect not observed with trust in family members, neighbors or strangers. These findings are consistent with the premise of relational cohesion theory and its extensions, which posit that individuals in environments lacking social cohesion often lack positive and successful social exchange behaviors and are accompanied by negative emotional reactions. As a result, these individuals are more susceptible to the negative impacts of interpersonal social interactions (Lawler and Yoon, 1996; Lawler et al., 2006). Conversely, in high-cohesion communities, individuals’ histories of positive, successful exchanges and mutual material or emotional support generate belonging-related positive emotions and heighten perceived control and security. These shared experiences cultivate collective efficacy, as residents believe they can jointly address challenges (Lawler et al., 2008; Nie et al., 2024; Van Bavel et al., 2020), which in turn buffers the adverse mental-health consequences of diminished interpersonal trust. Although the pandemic threat can trigger heightened vigilance, sensitivity, and distrust toward “others” in the environment (Fang et al., 2023; Nicomedes and Avila, 2020), individuals embedded in highly cohesive environments are more likely to adhere to cooperative norms highlighted by grassroots prevention initiatives and to engage actively in mutual aid and resource-sharing activities (e.g., emotional support, practical assistance), thereby mitigating pandemic-induced psychological stress effectively.(Cárdenas et al., 2023; Chan, 2021; Gibson and Losee, 2023; Townshend et al., 2015).

Furthermore, in line with the perspective provided by social capital theory, social cohesion, as a form of social capital generated from past experiences, can enhance residents’ confidence and optimistic expectations in overcoming current disasters (Drury et al., 2019; Ware, 2024; Miao et al., 2020). These individuals are better able to form cooperative relationships with others and adapt to their living environments (Uslaner, 2002). In summary, the threatening environment created by the pandemic, filled with perceived dangers, affected trust among people and, in turn, undermined residents’ psychological states. Conversely, in such situations, social cohesion within one’s living circle serves as a “protective” resource for residents, helping them better cope with uncertainties and life challenges (Bian et al., 2021; Hajek et al., 2023; Qin, 2021).

### 4.4 Limitations and future directions

This study presents a social-interaction framework that clarifies how perceived COVID-19 risk is associated with mental-health outcomes. However, several limitations inherent in the current study necessitate further exploration in future research endeavors. First, the present model focuses exclusively on the interpersonal pathways through which public-health events affect individuals’ physical and mental well-being. Future disaster-response research should therefore integrate the enduring influence of K-type trust differentiation linked to personal infection histories (Gambetta and Morisi, 2022) and systematically examine subgroup heterogeneity across China’s socio-economic landscape (e.g., urban vs. rural residence, age cohorts) to yield a more comprehensive account. Second, the “ripple effect” approach to measuring perceived COVID-19 risk, based on the proximity of confirmed cases, may oversimplify the multidimensional nature of risk perception, which includes factors like scientific understanding, self-efficacy, and emotional regulation (Slovic, 2010). While this measure was practical for a large-scale survey, it was not validated against objective infection rates or broader psychological risk scales due to data limitations. Future research should incorporate objective case data from public health records or validated risk perception instruments to enhance construct validity and ensure robustness. Third, the use of brief measures for key constructs, such as single-item measures for interpersonal trust and a two-item scale for social cohesion, poses potential limitations for construct validity and measurement precision. While these measures were chosen to minimize participant burden in a large-scale survey during a public health crisis and demonstrated acceptable reliability (e.g., Cronbach’s *α* = 0.789 for social cohesion), they may not fully capture the multidimensional nature of these constructs. Single-item measures, in particular, limit the ability to assess internal consistency and may introduce measurement error, potentially attenuating effect sizes. Although the Harman single-factor test indicated minimal common method bias, future research should employ multi-item, validated scales to enhance construct validity and precision, particularly for complex constructs like trust and social cohesion. Additionally, alternative measurement approaches, such as ecological momentary assessments, could provide richer data on dynamic social interactions during crises. Fourth, although data were collected nationwide, the sample’s geographic clustering, age profile, and educational composition diverge from the national population, and the use of a convenience sample limits generalization to clinical populations or to disasters beyond the COVID-19 context. Future studies should therefore replicate these findings with more diverse and representative samples to bolster external and ecological validity. Moreover, caution is warranted when extrapolating to non-pandemic settings, as the social-distancing mandates and heightened risk perception that uniquely characterize COVID-19 likely magnify the salience of interpersonal trust and social cohesion for mitigating psychological distress above levels observed in chronic or non-crisis circumstances. Fifth, the mediation and moderation models explained a relatively small proportion of variance in psychological distress (*R^2^* < 0.1), which may limit the interpretability of the findings. This low explained variance is consistent with the complexity of mental distress, which is influenced by multiple factors beyond perceived risk, trust, and social cohesion, such as individual personality traits and health status (Osimo et al., 2021). However, in the context of a public health crisis affecting millions, even small effect sizes can have meaningful population-level impacts. Future research should incorporate additional predictors to increase explanatory power and enhance model robustness. Sixth, this study provides a theoretical framework elucidating the pathways through which the perceived pandemic risk precipitated mental health issues from a social interaction perspective. However, the cross-sectional design precludes establishing temporal precedence, a critical requirement for causal inferences in mediation models. For instance, heightened mental distress may lead individuals to perceive elevated pandemic risk and exhibit diminished trust, thereby reversing the hypothesized causal direction. Future research should employ longitudinal designs to track changes in perceived risk, trust, social cohesion, and mental distress over time, or experimental designs to manipulate risk perception and observe its effects on trust and mental distress, thereby strengthening causal claims. Additionally, real-time online survey databases (Jaso et al., 2022) or virtual simulation experiments (Li et al., 2022) could enhance the reliability of these conclusions.

### 4.5 Practical implications

Despite the aforementioned limitations, our findings hold several practical implications. First, given the significant inverse association between perceived pandemic risk tied to proximity to infection sources and mental distress among residents in pandemic areas, governmental agencies should rigorously curtail transmission pathways while concurrently avoiding geographically concentrated notifications of nearby cases within feasible response capacities to mitigate the psychologically disruptive impact of the outbreak. Second, empirical evidence confirms that interpersonal trust mediates the association between perceived pandemic risk and mental distress. Consequently, enhancing interpersonal trust constitutes a proximate pathway for mental-health intervention. Public authorities should therefore systematically cultivate supportive intimate-bond networks as a core pillar of disaster response. While some indirect effects in our mediation model are small (e.g., indirect effects ranging from 0.002 to 0.007), their practical significance should be considered in the context of a public health crisis affecting millions of individuals. In a population as large as China’s, even small effect sizes can translate into substantial psychological impacts, particularly when compounded by prolonged exposure to pandemic-related stressors. For instance, the 15.434% mediation through family trust suggests that interventions targeting family dynamics could meaningfully reduce mental distress for a significant number of residents. Similarly, the smaller but significant effects through acquaintance and neighbor trust (5.652 and 4.348%, respectively) highlight the importance of community-based interventions to bolster trust and mitigate mental distress. These findings underscore the need for targeted policies that enhance interpersonal trust to achieve population-level mental health benefits during pandemics. Third, this study found that social cohesion moderated the relationship between acquaintance trust and mental health, as well as that between neighbor trust and mental health. This indicates that residents from areas of low social cohesion should be the focus of interventions, as they are more likely to develop mental health problems when their trust in surrounding individuals decreases. Targeted support and resources should be provided for these residents to build a stable social support system to help them effectively manage their mental states. By addressing these limitations and implementing the suggested practical implications, future research and interventions can more effectively address the issue of disaster threat perception and its impact on individual mental health.

## 5 Conclusion

To sum up, this study’s moderated mediation model examined the relationship between perceived pandemic risk and mental distress among residents in pandemic areas, testing the mediating roles of different types of interpersonal trust and the moderating role of social cohesion. Its findings show that perceived COVID-19 risk is positively correlated with mental distress As a general rule, interpersonal trust at various levels (except for trust in strangers) acts as a potential mediator. Moreover, social cohesion moderates the second stage of the mediation process involving neighbor and acquaintance trust. Specifically, residents in pandemic areas with higher social cohesion perception are better able to mitigate mental distress arising from decreased neighbor and acquaintance trust.

## Data Availability

The raw data supporting the conclusions of this article will be made available by the authors, without undue reservation.
