# Correction: The perceived COVID-19 pandemic risk and mental distress in China: the mediating role of interpersonal trust and the moderating role of social cohesion

**DOI:** 10.3389/fpsyg.2025.1714759

**Published:** 2025-11-12

**Authors:** Hao Zhou, Xiangqian Liu, Jing Wu, Chuanjing Liao, Shengyu Zhao

**Affiliations:** 1 Moral and Psychological Education Research Center, Zhejiang University of Finance and Economics, Hangzhou, China; 2 Department of Early Childhood Education, Faculty of Education, Shenzhen Polytechnic University, Shenzhen, China; 3 College of Education, Wenzhou University, Wenzhou, China; 4 School of Public Basic Studies, Wenzhou Polytechnic, Wenzhou, China

**Keywords:** COVID-19 pandemic, perceived risk, interpersonal trust, social cohesion, mental distress

There was a mistake in Table 2 as published. The column headings were missing; variables “trust (neighbors)” and “trust (f & c)” were misplaced. The corrected Table 2 appears below.

**Table 2:** Descriptive statistics and correlations among variables.

	***M ±SD***	**1**	**2**	**3**	**4**	**5**	**6**	**7**
1 Perceived risk	0.22 ± 0.82	1						
2 Trust (family)	3.71 ± 0.51	−0.043^**^	1					
3 Trust (neighbors)	2.96 ± 0.52	−0.015^*^	0.227^**^	1				
4 Trust (f & c)	3.07 ± 0.43	−0.023^**^	0.254^**^	0.588^**^	1			
5 Trust (strangers)	2.08 ± 0.72	−0.007	−0.032^**^	0.337^**^	0.283^**^	1		
6 Mental distress	1.40 ± 0.58	0.045^**^	−0.168^**^	−0.133^**^	−0.110^**^	−0.032^**^	1	
7 Social cohesion	3.60 ± 0.70	−0.007	0.142^**^	0.368^**^	0.242^**^	0.157^**^	−0.108^**^	1

*N* = *18,278*. f & c, friends and colleagues (acquaintance). ^**^p < 0.01; ^*^p < 0.05.

A correction has been made to section **3 Results**, *3.2 Preliminary analysis*, Paragraph 2. The descriptive text introducing Table 2 has been adjusted so that “trust (neighbors)” and “trust (f & c)” now appear in their correct locations:

“The correlation matrix (zero-order Pearson's correlation coefficients) is shown in Table 2. As expected, perceived pandemic risk is significantly positively correlated with mental distress (*r* = 0.045, *p* < 0.01). It is also significantly negatively correlated with family trust (*r* = −0.043, *p* < 0.01), neighbor trust (*r* = −0.015, *p* < 0.05), and acquaintance trust (*r* = −0.023, *p* < 0.01), while the negative correlation with stranger trust (*r* = 0.007, *p* > 0.05) is not significant. Additionally, family trust (*r* = −0.168, *p* < 0.01), neighbor trust (*r* = −0.133, *p* < 0.01), acquaintance trust (*r* = −0.110, *p* < 0.01), and stranger trust (*r* = 0.032, *p* < 0.01) are all significantly negatively correlated with mental distress. Furthermore, social cohesion is not significantly negatively correlated with perceived pandemic risk (*r* = −0.007, *p* > 0.05) but is significantly negatively correlated with mental distress (*r* = −0.108, *p* < 0.01). It is also significantly positively correlated with family trust (*r* = 0.142, *p* < 0.01), neighbor trust (*r* = 0.368, *p* < 0.01), acquaintance trust (*r* = 0.242, *p* < 0.01), and stranger trust (*r* = 0.157, *p* < 0.01).”

There was a mistake in Table 4 and its caption as published. The predictor labels have been realigned; Table 4 now contains “Trust (f & c)” as the mediator variable throughout all models.

The corrected Table 4 and its caption appear below.

**Table 4:** Testing the mediation effect of perceived risk on mental distress via trust (f & c).

**Predictor**	**Model 1 mental distress**		**Model 2b trust (f & c)**		**Model 3b mental distress**	
	***b***	***t***	***b***	***T***	***b***	***t***
Perceived risk	0.046	6.249^***^	−0.024	−3.103^**^	0.043	5.932^***^
Trust (f & c) trust					−0.107	−14.667^***^
Gender	0.052	3.478^***^	−0.070	−4.647^***^	0.045	2.993^**^
Age	−0.006	−11.897^***^	0.000	0.696	−0.006	−11.891^**^
*R* ^2^	0.011		0.002		0.022	
*F*	66.437^***^		10.907^***^		104.195^***^	

*N* = 18,278. Each column is a regression model that predicts the criterion at the top of the column. ^***^p < 0.001; ^**^p < 0.01; ^*^p < 0.05. f & c, friends and colleagues (acquaintance).

There was a mistake in Table 5 and its caption as published. The predictor labels have been realigned; Table 5 now contains “Trust (f & c)” as the mediator variable throughout all models.

The corrected Table 5 and its caption appear below.

**Table 5:** Testing the mediation effect of perceived risk on mental distress via trust (neighbors).

**Predictor**	**Model 1 mental distress**		**Model 2c trust (neighbors)**		**Model 3c mental distress**	
	***b***	***t***	***b***	***t***	***b***	***t***
Perceived risk	0.046	6.249^***^	−0.016	−2.185^*^	0.044	6.025^***^
Trust (neighbors)					−0.123	−16.736^***^
Gender	0.052	3.478^***^	−0.068	−4.599^*^	0.043	−2.933^**^
Age	−0.006	−11.897^***^	0.007	13.958^***^	−0.005	−10.205^**^
*R^2^*	0.011		0.013		0.026	
*F*	66.437^***^		77.608^***^		120.611^***^	

*N* = 18,278. Each column is a regression model that predicts the criterion at the top of the column. ^***^P < 0.001; ^**^P < 0.01; ^*^P < 0.05.

The original version of this article has been updated.

